# Gastric carcinoma: review of the results of treatment in a community teaching hospital

**DOI:** 10.1186/1477-7819-5-81

**Published:** 2007-07-20

**Authors:** Vincent H Heemskerk, Fanneke Lentze, Karel WE Hulsewé, Anton GM Hoofwijk

**Affiliations:** 1 Maaslandziekenhuis, Department of Surgery, Sittard, The Netherlands; 2 Academisch Ziekenhuis Maastricht, Department of Surgery, Maastricht, The Netherlands; 3 Atrium Medisch Centrum, Department of Surgery, Heerlen, The Netherlands

## Abstract

**Background:**

The aim of this study is to provide data on long term results of gastric cancer surgery and in particular the D1 gastric resection.

**Methods:**

In the period 1992-2004, 235 male and female patients with a median age of 69 and 70 years respectively, were included with a stage I through IV gastric carcinoma, of which 37% was stage IV disease. Whenever possible a gastric resection was performed. In case of obstructive tumour growth palliation was provided by means of a gastro-enterostomy.

**Results:**

Gastrectomy with curative intent was achieved in 50%, palliative resection in 22%, palliative surgery (gastro-enterostomy) in 10% and in 18% irresectability led to surgical exploration only. Patients in the curative intent group demonstrated a 47% survival after 5 years and up to 34% after 10 years. However metastases where seen in 32% of the patients after gastrectomy with curative intent. After palliative resection one year survival was 57%, whereas 19% survived more than 3 years. Overall postoperative morbidity and mortality rates were 40% and 13% respectively.

**Conclusion:**

Long term survival after surgery for gastric cancer is poor and is improved by early detection and radical resection. However, palliative resection showed improved survival compared to gastro-enterostomy alone or no resection at all which may be an effect of adjuvant therapy.

## Background

Gastric carcinoma remains one of the leading causes of cancer related deaths, although the worldwide incidence is declining [1]. Prognosis has improved only moderately with low overall survival rates. Surgical excision offers the only possibility for cure or long term survival [2]. Japanese surgeons were the first to implicate extended lymphadenectomy in an effort to reduce local recurrence and improve survival [3]. This included removal of 12 different lymph node stations grouped around the stomach and the arteries originating from the celiac trunk as well as resection of the spleen and pancreas tail, although splenectomy and pancreas tail resection is no longer mandatory when lymph nodes from these stations can be otherwise removed. This D2 resection has been compared with the D1 resection which entails only removal of the stomach and its directly surrounding lymph nodes. Although Japanese studies demonstrated increased survival with low morbidity and postoperative mortality, two large multi centre randomised controlled studies were unable to reproduce these results and demonstrated higher morbidity and mortality rates, this same conclusion was drawn by an extensive literature review [2,4,5]. The D1 resection is therefore still considered the standard treatment for gastric cancer surgery in most western countries.

In recent years the relationship between hospital volume and outcome has been studied for several types of surgical therapies including gastric surgery [6,7]. Although most papers report on short term outcomes, long term survival rates are at least as relevant but these types of data are scarce. When studying recent literature it seems mostly data from patients undergoing a gastric resection procedure are presented, although a substantial part of the patients with gastric cancer present at a stage when resection is not possible anymore. In order to provide better insight in the short and long term outcomes of the surgical treatment of gastric cancer we evaluated the results from patients treated during the last 12 years. Furthermore, we compared these results with those obtained from the literature.

## Methods

A total of 235 patients treated in our department for histologically proven gastric carcinoma between 1992 and 2004, were identified from a surgical database. This database was specifically designed for our surgical department to enable us to collect data prospectively on surgical interventions and register complications as well as others details in the postoperative period. This database includes demographic parameters, as well as data concerning therapeutic interventions and complications. Additional information such as tumour stage and pathology reports was gathered retrospectively from the individual patient files, most of which are already part of our electronic patient file registry that was started in the year 1999. When necessary the general practitioner was contacted for additional information.

Preoperative work-up consisted standard of gastroscopy, upper gastro-intestinal series and blood count. Computed tomography (CT) scan was only performed in 50 patients in cases where metastasis where suspected. However, the most recent national oncology guidelines, do not advise CT scan as standard preoperative diagnostic work-up. Patients were referred by the gastroenterologists.

Gastrectomy was performed using a standard D1 dissection [8]. Tumour type and location within the stomach were registered as well as histological grade. Pathologic staging was reported according to the TNM grading system [9]. Life table analysis was performed to evaluate survival in all patient groups.

Subgroup analysis was performed for tumour stage, type and location, different surgical interventions, age and co-morbidity. Statistical analysis was performed for survival data of the different surgical intervention groups using SPSS statistical software version 12.0.01 (Chicago, Illinois, USA).

## Results

### Patients

A total of 235 patients were included, 141 male and 94 female with a median age of 69 years (range 37–88) and 70 years (range 34–91) respectively, with a median follow-up of 11.9 months. Over 99% of the patients had symptoms related to gastric carcinoma, most often consisting of weight loss, pain and anorexia. Only 2 gastric cancers were detected in symptom free patients, both of which were operated upon. Co-morbidity was present in 138 patients (59%), various patients having more than form of co-morbidity. This consisted primarily of cardiovascular and pulmonary diseases, specifications of which are found in table 1.

**Table 1:** Comorbidity in gastric cancer patients

Comorbidity	No. of Patients	%
Cardiovascular	87	37
Pulmonary	24	10
Diabetes	19	8
Other carcinoma	20	9
Previous gastrointestinal surgery	18	8
Body Mass Index ≥ 30	12	5
Clotting disorder	2	1
Other	11	5

### Tumour

Most tumours were located in the antrum and corpus. Twelve percent was located in the cardia, and in 9% linitis plastica (whole stomach) was found (table 2). All tumours consisted of adenocarcinoma, the majority of which were high graded. More than a third of the entire population presented with stage IV disease.

**Table 2:** Patient, surgery and morbidity data

		No. of patients	%
Tumour location			

	Antrum	109	46
	Corpus	44	19
	Fundus	2	1
	Overlap	13	6
	Cardia	28	12
	Whole stomach	22	9
	Residual stomach	11	5
	Unknown	6	3

Tumour type			

	Adenocarcinoma	235	100

Stage			

	Ia	12	5
	Ib	30	13
	II	33	14
	IIIa	55	23
	IIIb	9	4
	IV	88	37
	Unknown	8	3

Surgery			

	Curative resection	118	50
	Palliative resection	51	22
	Palliative surgery	23	10
	Explorative surgery	43	18

Postoperative morbidity			

		93	40

Adjuvant therapy			

		37	16

In the subgroup of patients undergoing resection with curative intent disease stages were as follows: 10.7% stage Ia, 25.6% stage Ib, 24% stage II, 26% stage IIIa, 1.7% stage IIIb, 6.6% stage IV and in 5% stage could not be determined.

### Surgical procedure

If possible, patients in our population underwent a D1 resection (table 3). When radical resection was impossible, the other options were palliative resection (51 patients), gastrointestinal bypass (23 patients) or surgical exploration (43 patients). Postoperative morbidity occurred in 40% of all patients. We discriminated between direct surgical complications and secondary morbidity (table 4). Patients with preoperative co-morbidity were slightly more at risk for developing postoperative morbidity (42% versus 37%) compared to the other patients. When only surgical exploration was performed postoperative morbidity decreased to 21%.

**Table 3:** Surgical interventions in study population

	Resection type	No. of patients	%
Curative resection		118	50
	Total gastrectomy	34	14
	Partial gastrectomy	80	34
	Gastrectomy after former B1 resection	2	1
	Oesophagus-stomach	2	1
			
Palliative resection		51	22
	Total gastrectomy	16	7
	Partial gastrectomy	34	14
	Oesophagus-stomach	1	0
			
Palliative surgery		23	10
	Gastro-enterostomy	23	10
			
Explorative		43	18

**Table 4:** Surgical complications and morbidity in study population

Surgical complications	%	No. of patients	Secondary morbidity	%	No. of patients
Anastomotic leak	6.0	14	Cardiovascular	8.5	20
Wound infection	2.1	5	Pulmonary	9.8	23
Motility disturbance	3.4	8	Urinary tract infection	3.0	7
Gastrointestinal damage *	4.3	10	Other	9.8	23

* splenic puncture, gastric perforation, hemorrhage

Length of stay for surgical patients varied between 0 and 126 days with a mean of 13.7 days. Patients with co-morbidity were, on mean, hospitalised 3 days longer. Only patients in the explorative surgery group had a significantly shorter duration of admission (7 days).

### Surgeons

The majority of surgical procedures (200) were performed by a total of 6 gastrointestinal surgeons. One general surgeon performed 23 operations and the remaining 13 operations were performed by three different general surgeons during night time emergency procedures.

The mean of surgical procedures per (gastrointestinal) surgeon per year varied from 3 to 13. The most experienced surgeon performed a total of 87 procedures. Complication rate varying from minor to major was on average 40%. Only the most experienced surgeon had a percentage of 32. The general surgeon had a complication rate of 43%.

### Mortality

Perioperative mortality was defined as mortality within 30 days or, when still admitted beyond 30 days, in-hospital mortality. Perioperative mortality was 14% for the entire population. In the subgroup of patients undergoing surgery with curative intent, regardless of age, postoperative mortality was 13%. However, age was an independent risk-factor. Mortality rates for patients up to 75 years and patients over 75 years of age were 10% and 17% respectively. For resection with curative intent mortality rates for these age groups were 8% vs. 17% respectively, the former percentage approximating the Dutch gastric cancer trial data.

### Survival

**Figure 1:**
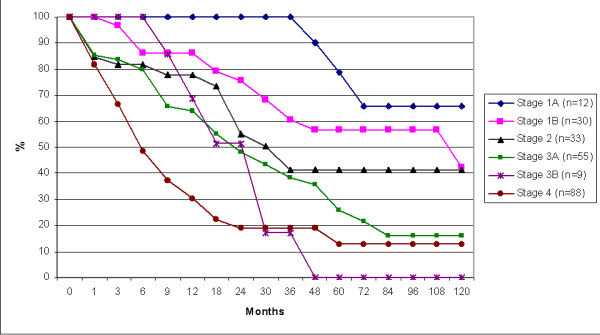
Survival in months of all gastric cancer patients divided per stage.

**Figure 2:**
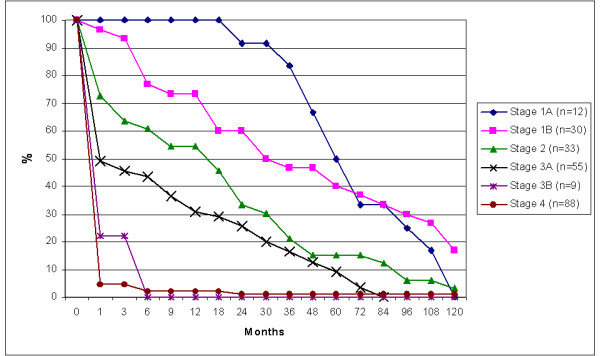
Disease free survival in months of all gastric cancer patients divided per stage.

**Figure 3:**
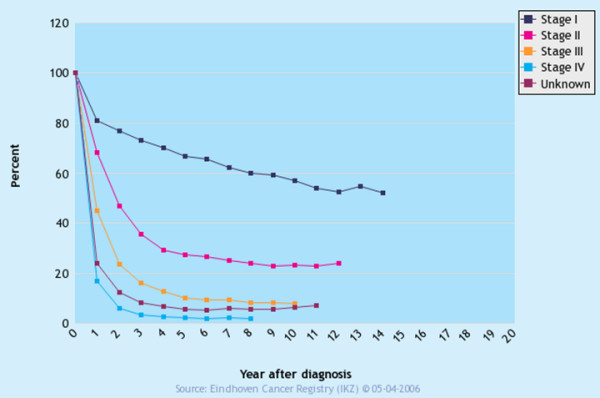
Survival in years of gastric cancer patients divided per stage in a neighbouring region in the Netherlands.

Overall survival ranged from 1 month to 142.5 months. When divided for different disease stages an obvious inverse relation between stage group and survival was detected (Figure 1; additional file 1) The 5 year survival for the different stages 1a, 1b, 2, 3a, 3b and 4 was: 79%, 57%, 41%, 26%, 0% and 13% respectively. However, disease free survival rates were substantially lower, also after prolonged follow-up periods (Figure 2 and additional file 1). The mean disease free survival for the different stages 1a, 1b, 2, 3a, 3b and 4 was: 60%, 40%, 15%, 9%, 0% and 1% respectively. In the Netherlands registration of survival of gastric carcinoma was so far only performed in two regions, the survival data from the neighbouring region are shown for comparison (Figure 3).

**Figure 4:**
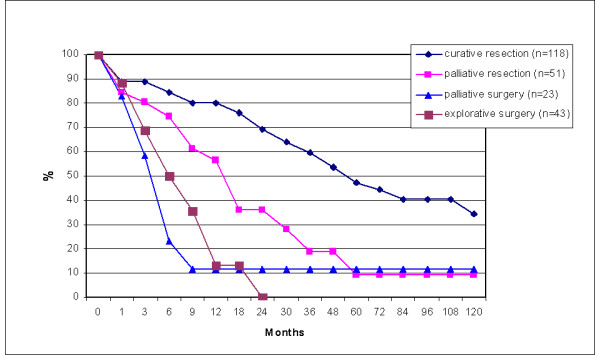
Survival in months of all gastric cancer patients divided per treatment group.

When focusing on surgical intervention, survival was highest in the curative resection group (N = 118) with a 10 year survival of 34% vs. 9% for palliative resection (N = 51). Palliative surgery (gastro-enterostomy, N = 23) showed a 12% survival over 10 years, whereas no patients survived surgical exploration (N = 43) over 2 years (Figure 4). Mean survival (±standard error) in the different surgical groups: curative resection, palliative resection, palliative surgery and explorative surgery was: 68%(±6), 25%(±6), 4%(±0.7) and 7%(±1) respectively.

Curative resection resulted in a significantly higher survival than palliative resection (p = 0.0007) and palliative resection in itself was better than gastro-enterostomy and explorative surgery (p = 0.0017) using the Wilcoxon (Gehan) test. During follow-up in 32% of the patients in the curative resection group metastases where detected.

Palliative resection resulted in a 57% 1-year survival, but decreased to 9% at 5 years (Additional File 2). No difference in survival was noted when determined for the various tumour locations, except for the whole stomach (linitis plastica) group, which carried the worst prognosis.

**Table 5:** Palliative resection vs no resection; patient characteristics

	Palliative resection (R1/R2)	No resection (GE/exploration)
no. of patients	51	66
median age (yr)	70	68
comorbidity (%)	63	47
complications (%)	39	21
adjuvant therapy (%)	31	18
no. of patients: male	34	43
no. of patients: female	17	23
admission days		
median (range)	12 (1–66)	8 (0–45)
30 day mortality (%)	16	20
6 months survival %	67	27
1 yr survival %	43	3
2 yr survival %	18	1
stage 1a (No. Of patients)	0	0
stage 1b (No. Of patients)	0	0
stage 2 (No. Of patients)	3	1
stage 3a (No. Of patients)	17	6
stage 3b (No. Of patients)	4	3
stage 4 (No. Of patients)	27	53
unknown stage	0	3

### Adjuvant therapy

Of all surgical patients included in this study, 37 patients (15%) received adjuvant therapy. This consisted of radiotherapy (11%), chemotherapy (76%), or a combination of both (13%). Of these patients 22 (59%) had a stage 4 tumour. Median survival after adjuvant therapy was 20 months.

## Discussion

The only possibility for curative treatment of gastric cancer remains surgical resection. However, patients typically present with an advanced stage as illustrated by our data where 37% of the population presented with stage 4 disease, resulting in only 50% of the patients to be eligible for surgery with curative intent. Furthermore, gastric cancer is more frequent in the older population (31% of the patients 75 years and older in our cohort) who have more co-morbidity and higher postoperative mortality rates [10] as confirmed by our data.

Survival after a D1 gastric resection with curative intent amounted to a 47% survival rate after 5 years and even a 34% survival after 10 years which demonstrates the importance of radical surgery. However, more extensive (D2) resection has not resulted in improved outcome in Western centres despite promising results from Japanese groups [3]. On the other hand, even in the curative surgery group, during longer follow-up 30% of the patients develop metastases. Apparently, (micro) metastases are often not recognized initially, ultimately leading to progressive disease. Although chemotherapy is not routinely applied in gastric cancer surgery, more studies are undertaken to explore it's possibilities and this may well improve disease free survival [11,12]. In our series for example, patients that lived longest (up to 50 months) belonged to the curative surgery group except for one patient that underwent palliative resection of a stage 3a tumour, combined with adjuvant chemotherapy, who survived up to 11 years without signs of metastases or recurrent disease.

Whereas data on the results of surgery for gastric cancer are available from several large multi centre studies, our results represent the outcome of the entire population treated surgically for stomach cancer. The selection bias in these larger studies probably explains for a large part the (relatively small) differences with the outcomes reported in this study. On the other hand, our data compare well with the long term results and stage dependent survival after gastric cancer surgery reported by the United States National Cancer Database [13] (table 6).

**Table 6:** Survival after gastric cancer surgery in our study compared to the US cancer database

Tumorstage	Survival at 5 years US (gastrectomy)	survival at 5 years in Sittard	number of patients per stage
Ia	78%	79%	12
Ib	58%	57%	30
II	34%	41%	33
IIIa	20%	26%	55
IIIb	8%	0%	9
IV	7%	13%	88

When curative resection was not possible, palliative resection was performed in a limited number of cases. Interestingly palliative resection seemed to improve survival compared to other palliative procedures, especially in the first postoperative year (Figure 4; additional file 2). However, these 2 palliatively treated groups (resection versus exploration or gastroenterostomy) differed with regard to tumour stage (less patients with stage 4 disease in the resection group) and appliance of adjuvant therapy (more frequent use of adjuvant chemotherapy in the resection group) (table 5). Which factor contributes most to this difference cannot be deduced from our data. The combination of cytoreduction and chemotherapy has been reported to result in a modest survival advantage [14]. Developments using hyperthermic intraperitoneal chemotherapy after cytoreduction show promising results, although residual tumour status remained the most important predictor of survival [15].

The other strategy to improve outcome of treatment for gastric cancer is to aim for early detection of gastric carcinoma. Unfortunately symptoms often present late in the course of the disease and usually are non-specific. Population based screening programs by means of gastroscopy has not been feasible in the western world due to low pre-test probability and relative high cost.

Zappa *et al*., combined a positive faecal occult blood test (FOBT) and a negative colonoscopy to select patients for gastroscopy in a large screening project. Still the number needed to screen was 254 without proven benefit on survival of the identified gastric cancer [16]. In a recent publication serum pepsinogen testing was reviewed to identify high risk patients, possibly combined with a helicobacter pylori test to reduce the amount of individuals that need gastroscopic screening [17].

Postoperative morbidity was high (40%) as this reflects all in hospital morbidity, both directly related to surgery, such as wound infections and anastomotic leakage, as well as postoperative morbidity in general, such as pulmonary or urinary tract infections. This figure is in line with results from other centres [18]. The underlying reasons are related to the fact that the population is relatively old with significant co-morbidities but nutritional depletion may also play a role. Therefore, careful attention should be paid to the perioperative care in this fragile patient group in order to improve outcomes.

## Conclusion

In conclusion, gastric cancer treatment is first and foremost favoured with early detection, as many studies have demonstrated stage dependent survival ratios. In general practice however, most patients are detected late in the course of disease as illustrated by the fact that only 50% of the patient in our series were operated with curative intent. However, the efficacy of screening programs needs to be improved prior to widespread implementation in western societies. Palliative resection, if possible, in combination with adjuvant chemotherapy may add to postoperative survival at least during the first year after surgery when compared with palliative surgery such as a gastro-enterostomy although this remains to be confirmed. Postoperative mortality and morbidity are relatively high and dictate optimal perioperative care.

Because the prognosis after surgery is determined primarily by the radicality of the resection, we believe that this type of surgery should be performed by dedicated surgeons. Further improvements in outcome probably can be expected by application of perioperative (neo) adjuvant therapies.

## Competing interests

The author(s) declare that they have no competing interests.

## Authors' contributions

VH participated in the study design, and contributed to data interpretation and analysis and writing the manuscript. FL contributed with study design, data acquisition, analysis of data and writing. KH participated in the study design and writing as well as revision of the manuscript for critically important intellectual content. AH participated in study design, data analysis and interpretation, critical manuscript revision and gave final approval

All authors read and approved final manuscript.

## Supplementary Material

Additional file 1 Micorsoft excel file showing Data to Figures 1 and 2 Click here for file

Additional file 2 Microsoft excel file showing Data to figure 4 Click here for file
